# Nanoparticle interactions with live cells: Quantitative fluorescence microscopy of nanoparticle size effects

**DOI:** 10.3762/bjnano.5.248

**Published:** 2014-12-11

**Authors:** Li Shang, Karin Nienhaus, Xiue Jiang, Linxiao Yang, Katharina Landfester, Volker Mailänder, Thomas Simmet, G Ulrich Nienhaus

**Affiliations:** 1 Institute of Applied Physics, Karlsruhe Institute of Technology (KIT), 76131 Karlsruhe, Germany; 2 State Key Laboratory of Electroanalytical Chemistry, Changchun Institute of Applied Chemistry, Chinese Academy of Science, Changchun, 130022, China; 3 Max Planck Institute for Polymer Research, 55128 Mainz, Germany; 4 Institute of Pharmacology of Natural Products & Clinical Pharmacology, Ulm University, D-89081 Ulm, Germany; 5 Institute of Toxicology and Genetics (ITG), Karlsruhe Institute of Technology (KIT), 76131 Karlsruhe, Germany; 6 Department of Physics, University of Illinois at Urbana-Champaign, Urbana, Illinois 61801, USA

**Keywords:** cell membrane, endocytosis, fluorescence microscopy, nanoparticle, size effect

## Abstract

Engineered nanomaterials are known to enter human cells, often via active endocytosis. Mechanistic details of the interactions between nanoparticles (NPs) with cells are still not well enough understood. NP size is a key parameter that controls the endocytic mechanism and affects the cellular uptake yield. Therefore, we have systematically analyzed the cellular uptake of fluorescent NPs in the size range of 3.3–100 nm (diameter) by live cells. By using spinning disk confocal microscopy in combination with quantitative image analysis, we studied the time courses of NP association with the cell membrane and subsequent internalization. NPs with diameters of less than 10 nm were observed to accumulate at the plasma membrane before being internalized by the cells. In contrast, larger NPs (100 nm) were directly internalized without prior accumulation at the plasma membrane, regardless of their surface charges. We attribute this distinct size dependence to the requirement of a sufficiently strong local interaction of the NPs with the endocytic machinery in order to trigger the subsequent internalization.

## Introduction

Understanding the interaction between engineered nanomaterials and living matter has attracted increasing attention in recent years, especially in view of possible implications regarding biosafety and biomedical applications of nanomaterials [1–5]. Because NPs have sizes similar to those of biological molecules and assemblies such as proteins or viruses, they are able to invade cells by hijacking the cellular endocytosis machinery. An interesting aspect in this process is that, in the biological milieu, the NPs typically adsorb dissolved biomolecules, so that they are enshrouded by a so-called ‘protein corona’ [6–8]. NPs interact with cells via this layer of biomolecules, at least during the initial encounter, so that it determines the biological identity of the NP.

**Figure 1:**
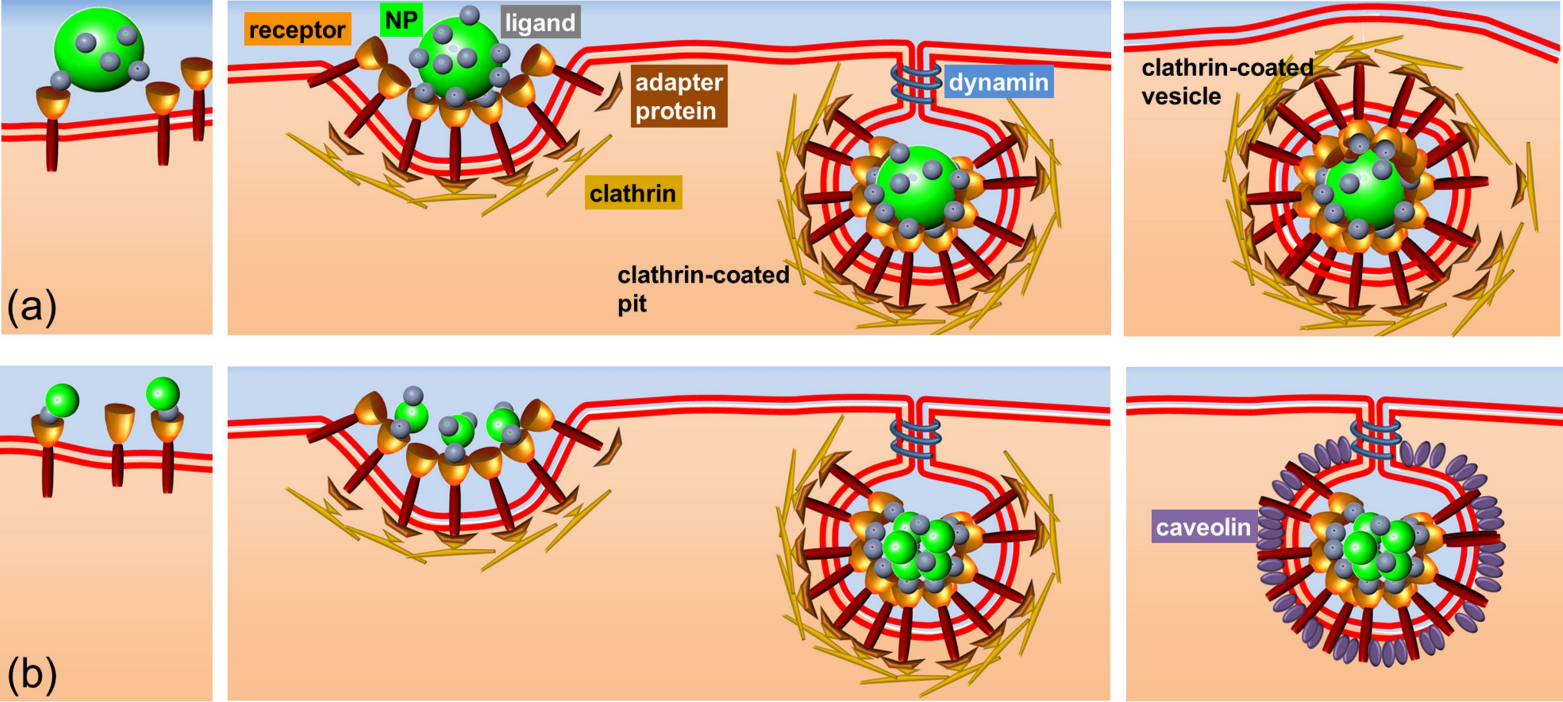
Schematic representation of the cellular uptake of (a) large and (b) small NPs. Whereas larger NPs exert interactions with the cell membrane that are sufficiently strong to trigger internalization of one NP at a time, smaller NPs have to form a cluster of a certain size to induce membrane invagination.

The key role of a cellular membrane is to provide a strict separation between the cytosol and the extracellular environment, and to selectively control the flow of ions and molecules into and out of the cell. For internalization of larger chunks of material, e.g., lipoprotein particles, protein assemblies, viruses and NPs, these are typically encapsulated in vesicles and selectively transported into and out of the cells via endocytosis and exocytosis, respectively [9–10]. Depending on the size of the transport vesicle, cargo properties and the internalization machinery involved, different endocytic mechanisms are utilized. Most cells are capable of pinocytosis (drinking by cells), in which particles of up to several hundred nanometers can be internalized [11]. In this process, an invagination forms in the cell membrane. Typically, the inward budding vesicle contains receptor proteins that recognize specific chemical groups on the biomolecules to be internalized. It is finally pinched off so as to generate a vesicle in the cytoplasm that contains the internalized material (Figure 1). Different pinocytosis mechanisms are being distinguished, depending on the specific uptake machinery involved, these are macropinocytosis, clathrin- and caveolae-mediated endocytosis, and mechanisms that involve neither clathrin nor caveolae. The exposed functional groups on an NP surface interact with cell surface receptors and may activate the cell’s uptake machinery. Depending on the details of their interactions, proteins adsorbed onto NPs may enhance or reduce internalization of the so disguised NPs.

Specialized cells, such as macrophages, neutrophils, and monocytes are capable of phagocytosis (eating by cells), a form of endocytosis in which the cell internalizes larger, typically micron-sized particles by completely engulfing them with their plasma membrane. In addition to intruding cells by active transport, NPs may also enter cells by passive membrane penetration. In fact, cell types that completely lack the endocytosis machinery such as red blood cells (RBCs) can internalize NPs only via passive transport [12].

The efficiency of NP internalization by a cell depends on cell-specific parameters such as cell type or cell cycle phase [13–14] and physicochemical properties of the NP [15]. Notably, NPs with positive surface charge are typically incorporated by cells to a larger extent than negatively charged ones, owing to their stronger coulombic interactions with the negatively charged plasma membrane [16–18]. Also, spherically shaped NPs have been reported to enter cells more efficiently than non-spherical (i.e., rod-like) NPs, which may be related to the different curvature of the adsorbed NPs experienced by the cell [19]. Apart from shape and surface charge, the NP size plays a crucial role in modulating the NP-cell interactions [20]. It affects the uptake efficiency and kinetics, the preference for certain internalization pathways as well as the subcellular distribution upon internalization [21–23].

Presently, the effect of NP size on cellular uptake is discussed controversially, which may, at least in part, be associated with the diverse experimental conditions and techniques chosen to monitor NP-cell interactions on cultured cells [20]. Typically, live cells are immersed in a medium supplemented with blood serum to ensure cell viability. NPs in the medium form a biomolecular adsorption layer that depends on the composition of the medium. Therefore, the outcome of these experiments strongly depends on the choice of medium [24–27]. Because of the inevitable protein corona formation in any biological environment, one has also to be aware that experiments on cultured cells may yield results different from in vivo studies.

Over the past few years, we have used spinning disk confocal fluorescence microscopy to systematically quantify the uptake kinetics of fluorescent NPs in the size range of 3.3–100 nm (diameter) by live cells. This imaging technique is non-invasive and, because of its high temporal and spatial resolution, well suited to watch NPs invade cells in real time. Here we compare the cellular uptake of NPs with widely differing sizes. We have selected very small gold nanoclusters (AuNCs, diameter ≈3 nm) stabilized with dihydrolipoic acid (DHLA), semiconductor core-shell quantum dots (CdSe/ZnS, ≈10 nm) coated with D-penicillamine (DPA) and relatively large polystyrene (PS) NPs (≈100 nm) with different surface functionalizations and investigated their interactions with various human cell lines, in particular HeLa cells and mesenchymal stem cells (MSCs). Of note, these studies were carried out in phosphate buffered saline (PBS), pH 7.4, or serum-free DMEM, so that we could probe interactions between cells and the bare NP surfaces rather than NPs carrying a protein corona of unknown composition. By means of inhibitory drugs that specifically interfere with the one or the other endocytosis pathway, the endocytosis pathways involved in the uptake of small and large NPs have been revealed. Finally, some general conclusions are drawn as to how the NP size affects the mechanistic details of the uptake process.

## Results and Discussion

### NP characterization

We have synthesized differently sized, water-soluble NPs, including intrinsically luminescent D-penicillamine-coated quantum dots (DPA-QDs) [28], dihydrolipoic acid-coated gold nanoclusters (DHLA-AuNCs) [29], and fluorescently labeled polystyrene (PS) NPs with covalently attached carboxyl (–COOH, CPS) or amine (–NH_2_, NPS) surface functionalizations [30]. For comparison, we have also studied plain PS NPs, which were water-solubilized by physically adsorbed amphiphiles, the anionic surfactant SDS or cetyltrimethylammonium (CTMA) chloride to yield negatively (PS^−^) and positively (PS^+^) charged NPs, respectively. The hydrodynamic diameters of these NPs suspended in PBS, pH 7.4, were determined by dynamic light scattering (DLS) (Table 1). Furthermore, the zeta potentials of all NPs in PBS were also measured. As expected from the surface chemistry of the NPs, the NH_2_-modified (NPS) and the CTMA-adsorbed NPs carried a positive surface charge; all other preparations had a negative surface charge.

**Table 1:** Size and surface charge characterization of NPs employed in this study.

NP	hydrodynamic diameter (nm)^a^	zeta potential (mV)^b^

DHLA-AuNCs^c^	3.3 ± 0.3	−(37 ± 3)
DPA-QDs^d^	8.0 ± 0.6	−40
PS^−^ NPs^e^	116 ± 7	−(45 ± 5)
CPS NPs^e^	122 ± 9	−(46 ± 6)
PS^+^ NPs^f^	100 ± 5	+(50 ± 8)
NPS NPs^f^	113 ± 6	+(59 ± 10)

^a^Determined from the number distribution of DLS data. ^b^Measured using a Malvern Zetasizer (Malvern Instruments, Malvern, UK). Data taken from ^c^Ref. [28], ^d^Ref. [31], ^e^Ref. [32], ^f^Ref. [33]

### Cellular uptake of small (diameter 3–10 nm) NPs

**Figure 2:**
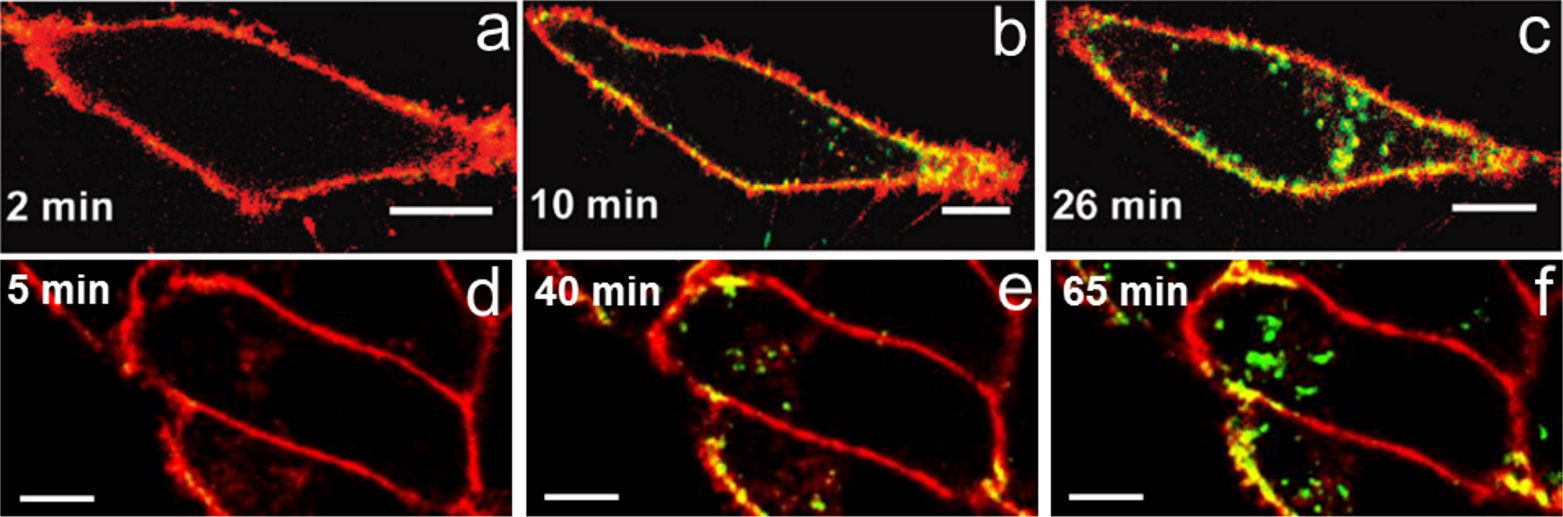
Typical two-color merged confocal fluorescence microscopy images of live HeLa cells exposed to NPs (green); times after the start of NP incubation is given in the panels. (a–c) DPA-QDs, 10 nM in PBS; (d–f) DHLA-AuNCs, 20 µg/mL in serum-free DMEM. Scale bar, 10 µm. Cell membranes (stained with CellMask DeepRed) are depicted in red. Images in panels a–c were reproduced with permission from [31]. Copyright 2010 American Chemical Society. Images in panels d–f were reproduced with permission from [34]. Copyright 2013 Royal Society of Chemistry.

Figure 2 shows representative two-color merged fluorescence images recorded at selected times during the exposure of cultured HeLa cells to DPA-QDs in PBS and DHLA-AuNCs in DMEM solution. The cell membrane and the NPs are depicted in red and green color, respectively; colocalization is shown in yellow. Within 5 min, both DPA-QDs and AuNCs started to accumulate at the plasma membrane. With increasing exposure time, NPs also appeared in the intracellular region, where they formed large clusters.

**Figure 3:**
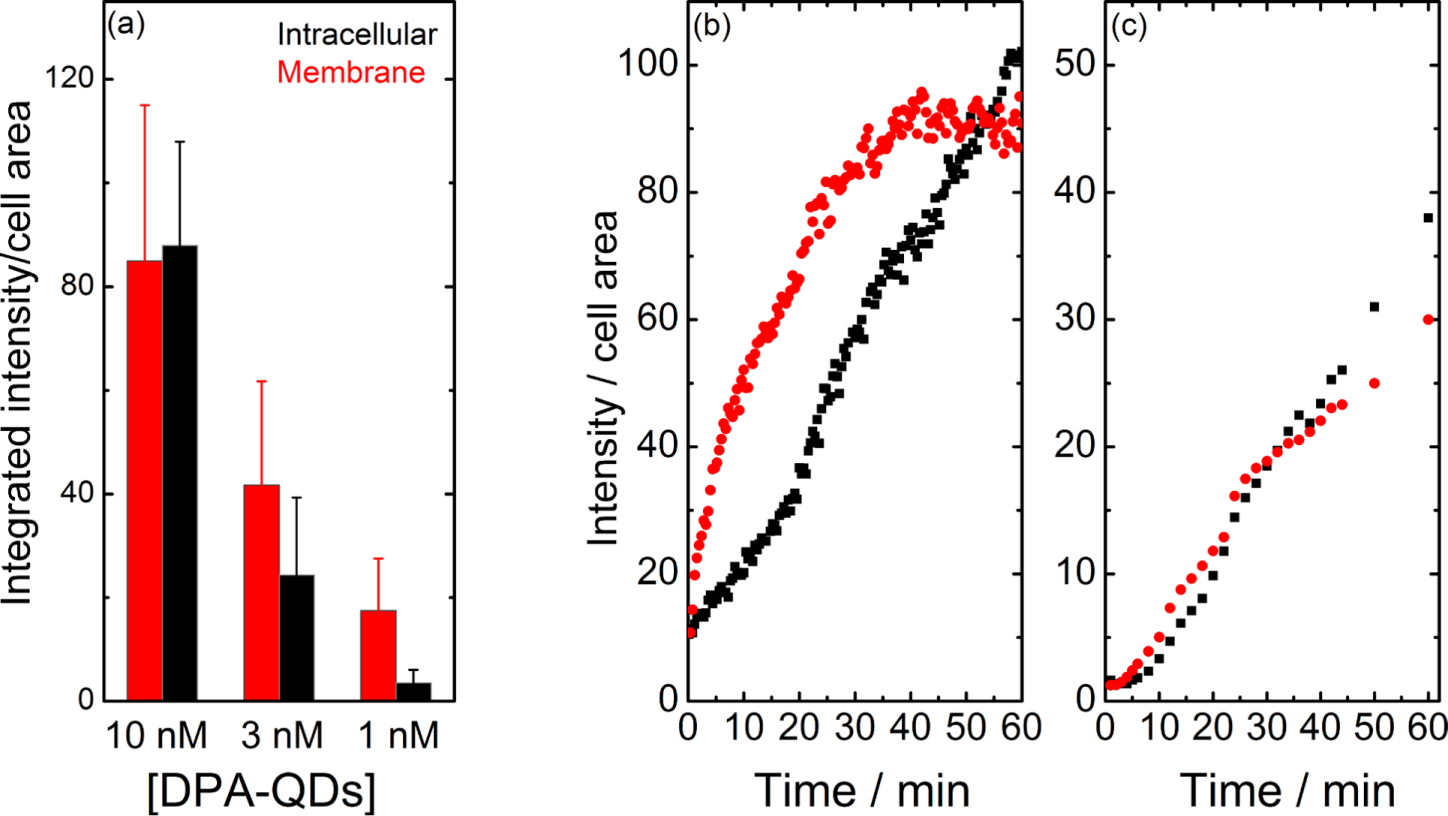
(a) DPA-QD uptake within 1 h by live HeLa cells as a function of NP concentration. (b) DPA-QD and (c) AuNC accumulation in the intracellular (black symbols) and membrane (red) regions of live HeLa cells within the first 60 min. Cells were exposed to (a, b) 10 nM DPA-QDs and (c) 1 µM DHLA-AuNCs. Images in panels a and b were reproduced with permission from [31]. Copyright 2010 American Chemical Society; the image in panel c was reproduced with permission from [34]. Copyright 2013 Royal Society of Chemistry.

To study the time dependence of NP membrane association and internalization by exposing cells to DPA-QDs at varying concentrations (1–10 nM), fluorescence microscopy was performed over time courses of typically 1–2 h. Quantitative analysis of the image sequences revealed that the amount of NPs associated with the membrane scaled, within the error, with the NP concentration in solution (Figure 3a). The fraction of internalized NPs, however, decreased much more strongly with decreasing NP concentration (Figure 3a). Strong accumulation on the membrane within the first few minutes, observed at DPA-QD concentration of 10 nM, was followed by fast, continuous internalization. By contrast, even after a 1 h exposure to a 1 nM DPA-QD solution, DPA-QDs were barely detectable inside the cells. A quantitative analysis of the DPA-QD uptake kinetics confirmed that the NPs accumulated on the membrane before uptake occurred (Figure 3b). The same effect was also observed for AuNCs (Figure 3c). These findings clearly indicate that a certain density of small NPs on the plasma membrane is required to initiate cellular internalization; an individual small NP is not capable of triggering endocytosis by itself.

### Cellular uptake of large (diameter ≈100 nm) NPs

We have also systematically investigated the uptake of ≈100 nm PS NPs by mesenchymal stem cells (MSCs) in PBS solution. Because the initial step of endocytosis, i.e., the encounter of the NP with the cell membrane, is expected to be sensitive to the NP surface functionalization, we have exposed MSCs to two types of well-defined, positively charged PS NPs with essentially the same size and surface charge, namely plain, CTMA-stabilized PS^+^ NPs and amino-functionalized PS NPs (NPS NPs) carrying about 6,000 amino groups on their surfaces (Table 1) [33]. This study was further extended to include the effect of surface carboxyl groups on the interaction of anionic NPs with MSCs. Therefore, negatively charged, SDS-stabilized PS^−^ NPs and those with covalently bound carboxylic acid groups (CPS NPs) were prepared [32]. These likewise had the same size and surface charge (Table 1).

**Figure 4:**
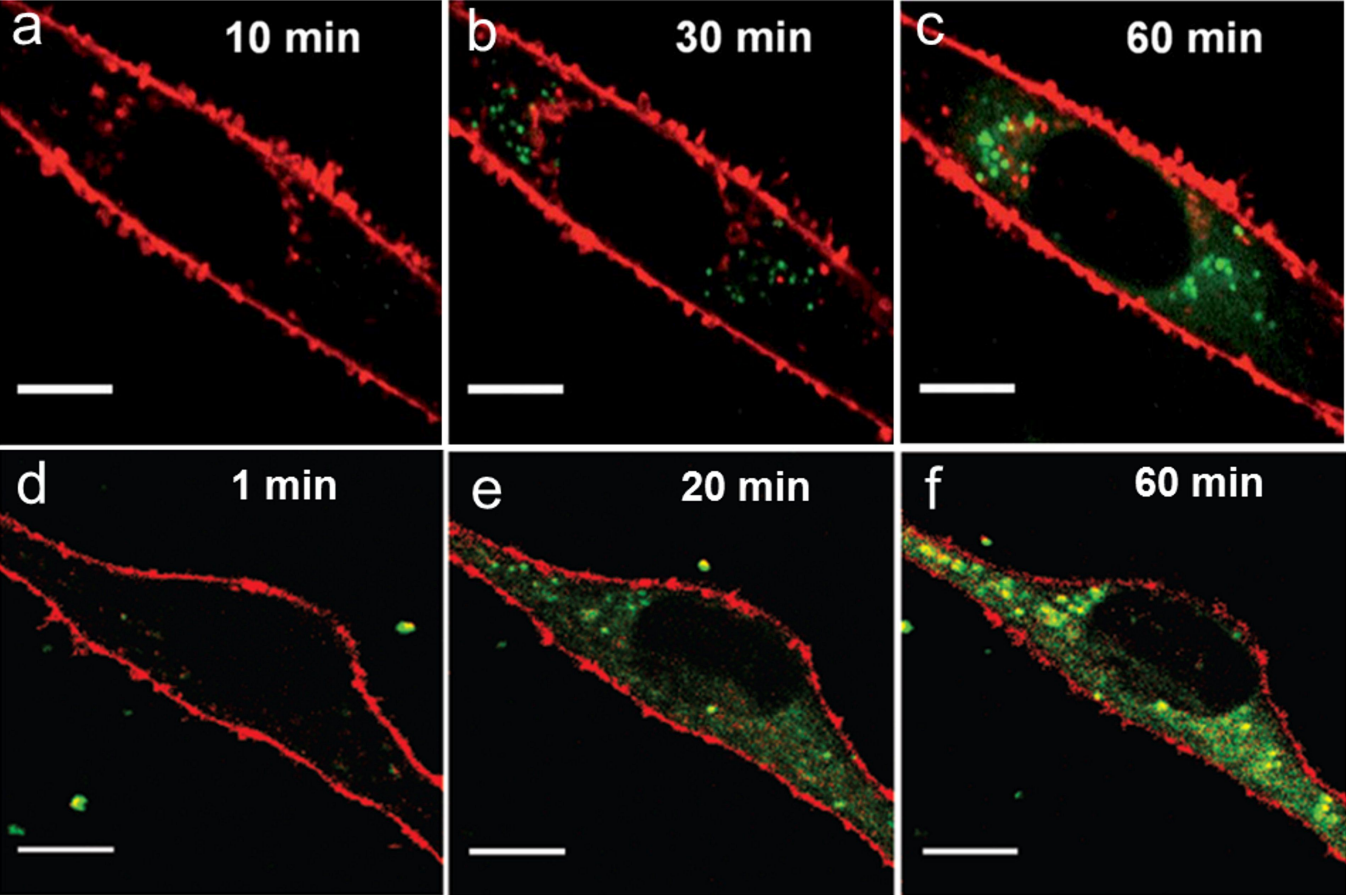
Two-color merged confocal images of live human MSCs exposed to NPs (green) in PBS for different times. (a–c) PS^−^ NPs, 75 µg/mL; (d–f) PS^+^ NPs, 7.5 µg/mL. Scale bar, 10 µm. Cell membranes (in red) are stained with CellMask DeepRed. Images in panels a–c were reproduced with permission from [32]. Copyright 2011 Royal Society of Chemistry. Images in panels d–f were reproduced with permission from [33]. Copyright 2010 American Chemical Society.

Figure 4a–c shows typical fluorescence images of MSCs after exposure to anionic PS^−^ NPs (75 µg mL^−1^) in PBS for selected times. After ≈10 min, internalization of PS^−^ NPs was noticeable. Subsequently, the NPs gradually accumulated within the cell, as seen from the continuous increase of bright fluorescent spots inside the cell. In contrast to the uptake studies on the smaller DPA-QDs and AuNCs (Figure 2), there was no bright fluorescence visible at the cell membrane, however, indicating that the large PS^−^ NPs were rapidly endocytosed without accumulating on the plasma membrane. Otherwise, their presence would have caused yellow spots to appear in the overlay image of pseudo-colored red membrane and green NPs. For cationic PS^+^ NPs of similar size, much faster uptake by the MSCs was observed (Figure 3d–f), with NPs appearing in the cell as early as 1 min after being added, despite being administered at ten-fold lower concentration than the anionic NPs. Compared with the plain anionic PS^−^ NPs, cellular internalization (within 1 h) of CPS NPs was ≈5-fold more effective, with only few NPs visible near the plasma membrane. NPS NPs also showed a higher intracellular intensity than the control non-functionalized PS^+^ NPs; the difference was less pronounced, though.

The much lower uptake rate of anionic PS NPs in comparison to cationic PS NPs suggests that the surface charge strongly facilitates their approach to the cell surface [17,35–36]. The negative potential of the cell surface repels negatively charged NPs. However, once an NP has reached the cell membrane, specific functional groups on the NP surface, e.g., carboxyl groups, overcome the Coulomb repulsion and facilitate binding to membrane-bound receptors that activate the endocytosis machinery. However, independent of the surface charge and functionalization, NP accumulation on the membrane was entirely absent during uptake of 100 nm NPs. Thus, we conclude that, upon binding to the cell membrane, these large NPs are immediately endocytosed.

### Elucidating NP uptake pathways

Endocytosis enables cells to internalize nutrients, including macromolecules and larger particles. The choice of the internalization mechanism may depend on the properties of the cargo. For example, clathrin-dependent endocytosis is an important entry route of NPS into mammalian cells. The relative importance of the different mechanisms can be studied by selective application of inhibitors that can specifically suppress particular pathways [37–39]. Dynasore, e.g., suppresses dynamin-dependent endocytosis pathways, including clathrin- and caveolin-mediated endocytosis [40]. The multidomain GTPase dynamin usually forms a helix around the neck of a nascent vesicle and, by axially extending this helix via GTP hydrolysis, leads to pinching and release of the vesicle from the parent membrane into the cytosolic compartment (Figure 1). This process is inhibited by dynasore. Clathrin creates a polyhedral lattice around newly forming vesicles and associates with the receptors in the membrane (that anchor the cargo) through adaptor proteins to assemble a clathrin-coated pit (Figure 1). Clathrin assembly at the plasma membrane and, therefore, pit formation is suppressed in the presence of chlorpromazine [41]. In Table 2, we have summarized the qualitative effects of dynasore and chlorpromazine on NP uptake.

**Table 2:** Effects of inhibitors on NP uptake^a^.

NP / surface charge / cell type			dynasore			chlorpromazine		

			Total	Membr.^b^	Intracell.^c^	Total	Membr.	Intracell.

≈100 nm:								

PS^+^ NP^d^	+	MSC	–	–	–	0	0	0
NPS NP^d^	+	MSC	–	(–)	–	–	–	–
PS^−^ NP^e^	–	MSC	0	0	0	0	0	0
CPS NP^e^	–	MSC	–	–	–	–	–	–

≈10 nm:								

DPA-QD^f^	–	HeLa	–	0	–	–	–	–
DHLA-AuNC^g^	–	HeLa	–	(–)	–	–	–	–

^a^(–): (minor) decrease compared to control without inhibitor; 0: no inhibitory effect. ^b^membrane-associated fraction. ^c^intracellular fraction. Data taken from ^d^Ref. [33], ^e^Ref. [32],^f^Ref. [31], ^g^Ref. [34].

From the data in Table 2, it is apparent that the uptake efficiency of small NPs in HeLa cells is affected by both dynasore and chlorpromazine [31,34]. Chlorpromazine reduced both the membrane-associated and the intracellular fractions. Because chlorpromazine disturbs clathrin-coated pit formation, it lowers both the binding capacity of the plasma membrane and vesicle internalization. Dynasore, however, had no measurable effect on the membrane-associated fractions of both DPA-QDs and DHLA-AuNCs. These results make sense, considering that dynasore suppresses the pinching-off process but not the formation of clathrin-coated pits. As long as internalized vesicles are continuously replaced by newly forming ones, the overall number of NP binding sites on the membrane in steady state should not be significantly affected by dynasore. Taken together, our inhibitor studies provided evidence that our small NPs are internalized via clathrin-dependent pathways. In agreement with our results, other studies also showed that clathrin-dependent uptake plays an important role in the internalization of nanoparticles*,* e.g., silver NPs (diameter: 50 nm) [42] and polystyrene NPS (diameter: 40 nm) [43].

The presence of dynasore also reduced the uptake of 100 nm CPS NPs in MSCs by ≈70%, whereas no effect was observed for PS^−^ NPs under the same conditions [32]. These results suggest that the carboxylic acid groups on the NPs caused a strong preference for dynamin-dependent endocytosis. Dynasore also reduced the uptake of positively charged PS NPs, independent of their functionalization, which may indicate that their uptake is mainly governed by the overall charge and not so much by the specific functionalization [33], most likely because of the favorable coulombic interactions between the oppositely charged interaction partners.

In the presence of chlorpromazine, the uptake of NH_2_-PS NPs was suppressed by ≈70%, whereas little effect was seen for PS^+^ NPs. The uptake of negatively charged CPS NPs, but not of non-functionalized PS^−^ NPs, was also reduced. Apparently, both the carboxyl and the amino functional groups favor clathrin-mediated uptake, possibly via a high affinity to their receptors.

Lunov et al. [44] investigated the uptake of CPS and NPS NPs by human macrophages and by undifferentiated and PMA-differentiated monocytic THP-1 tumor cells in great detail and could show that uptake of the same kind of PS NPs into different cells lines occurred via diverse mechanisms. The amount of internalized NPs, and also the uptake kinetics, differed considerably between primary cells and a related tumor cell line, whether differentiated or not. The uptake mechanism associated with a particular NP preparation did not only depend on the cell line but also on whether internalization was analyzed with the cells in buffer or in medium containing human serum, so that a protein adsorption layer will form on the NPs. These data emphasize that studies of NP-cell interactions on cell line models may not be straightforwardly transferable to the situation in normal differentiated cells. They also found that only the NPS NPs triggered NLRP3 inflammasome activation and subsequent release of proinflammatory interleukin 1β (IL-1β) by human macrophages [45].

Overall, these studies stress that cellular uptake pathways crucially depend on specific interactions of the NPs with cell surface receptors, which subsequently activate different pathways. Markedly different mechanisms can be involved in the endocytosis of NPs with identical size and surface charge but different surface functionalities.

### Mechanistic details of size-dependent NP uptake

**Figure 5:**
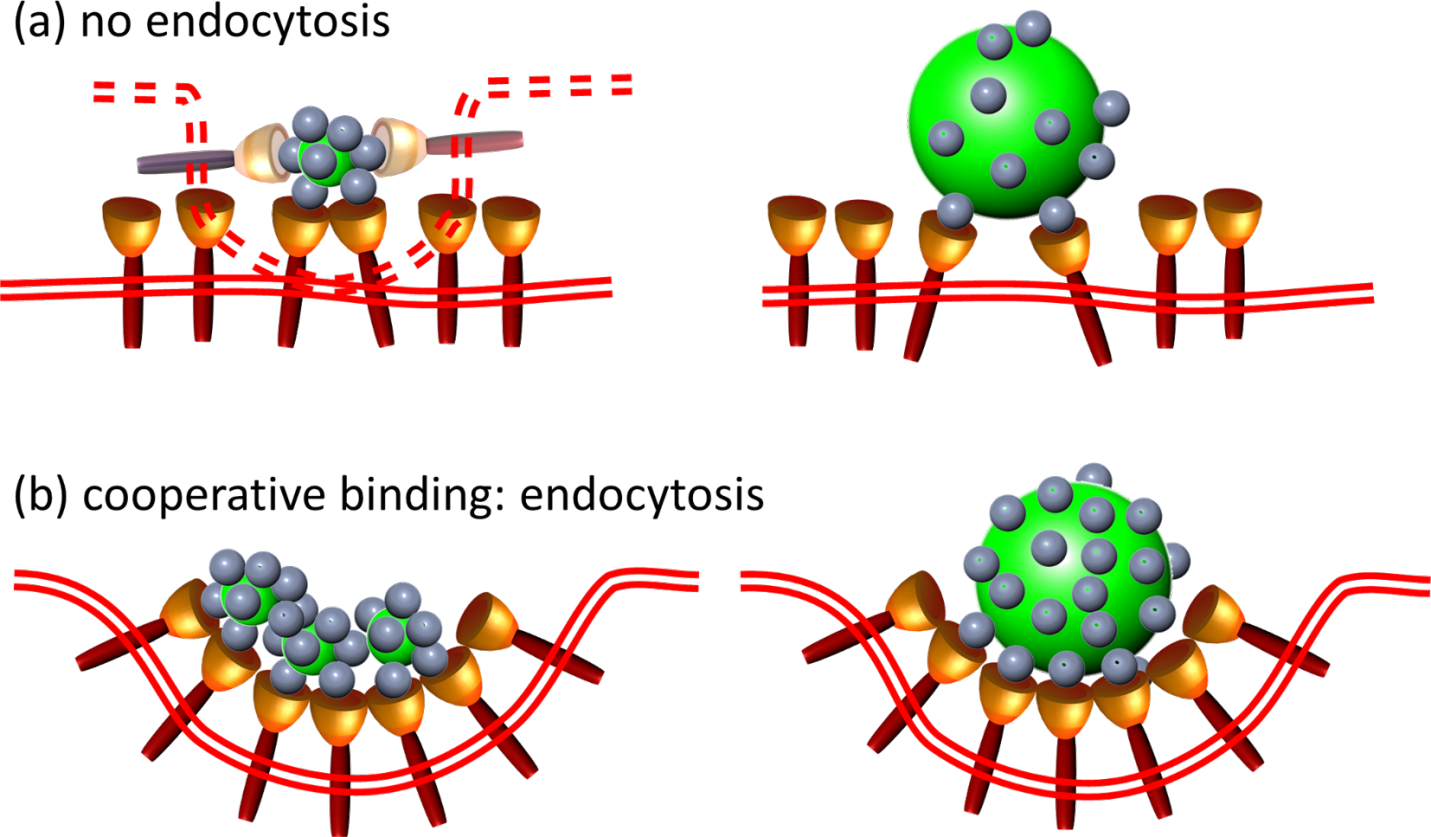
Schematic depiction of the interplay between membrane receptors (depicted as cups) and NP surface ligands (depicted as gray spheres). (a) The number of ligands offered to the receptors is insufficient to induce invagination, either because (left) the NP or (right) the density of functional groups is too small. To allow for a sufficiently large number of ligand–receptor interactions, an extreme (and unrealistic) membrane curvature (dashed) would be required. (b) The number of successful receptor interactions can be increased by either clustering small NPs or having high densities of functional groups on larger NPs.

Our observations clearly show that NP size plays an important role in their interactions with cells during the endocytosis process. However, size should not be discussed uncoupled from the surface functionalization. In fact, an NP can be considered as a scaffold carrying ligands that interact (or avoid the interaction) with receptor targets anchored in the cell membrane (Figure 5). The number of available ligands depends on the ligand grafting density on the NP but also on the NP curvature.

Receptor-mediated endocytosis is a complicated process that can be facilitated by a variety of proteins such as clathrin or caveolin. Mechanistic models point to the crucial role of NP-receptor interactions in the formation of endocytic vesicles [46]. The internalization process starts by binding of NP surface ligands to receptors on the cell membrane. The thermodynamic driving force is mainly controlled by the receptor and ligand densities and the receptor-ligand binding energy. It competes with the energy cost required to bend the membrane, which depends on the membrane tension and the NP curvature and, therefore, on the NP size. If the overall energy balance is equivalent to a localized decrease in the Gibbs free energy, the membrane will wrap around the NP. A vesicle-like structure is formed that eventually buds off and fuses with other vesicles to form endosomes [46–47].

Such a mechanistic model allows us to understand the size dependence of NP internalization. A small NP can interact only with one or two membrane receptors so that small NPs have to work in a cooperative way, i.e., by locally clustering in close proximity to each other (Figure 5), to recruit and bind enough receptors to successfully drive membrane wrapping to induce internalization. Our NP uptake experiments (Figure 2) revealed that small QDs or AuNCs (diameter less than 10 nm) accumulate at the membrane prior to internalization by the cell as a whole (Figure 3), and that a certain NP concentration on the membrane is required to overcome the energy costs associated with membrane bending.

By contrast, a larger NP can act as a cross-linking agent capable of clustering nearby receptors (Figure 5). Therefore, it can easily be enwrapped without the involvement of neighboring NPs. From a thermodynamic view of point, it was reported that a 40–50 nm NP binds enough receptors to produce sufficient membrane wrapping [47]. Particles smaller than the critical size of ≈50 nm are likely to be packaged as a cluster in one vesicle with the optimal size. Of note, Aoyama and colleagues [48] studied size effects and receptor contributions in glycoviral gene delivery and concluded that receptor-mediated endocytosis is optimal for ≈50 nm artificial glycoviruses.

Based on studies of the uptake of carboxydextran-coated superparamagnetic iron oxide NPs of 20 and 60 nm by human macrophages, Lunov et al. [49] developed a mathematical model that predicts the wrapping times of different NPs. In addition, the relation between membrane elasticity, cytoskeletal forces and the uptake time is described, which allows the determination of key parameters of endocytosis, such as the uptake rate, the saturated number of NPs per cell, the mean uptake time as well as the correlation between the number of internalized NPs and their extracellular concentration.

Gao et al. [46] suggested that NPs in the size range of tens to hundreds of nanometers can enter cells via wrapping even in the absence of clathrin or caveolin coatings. Upon contact with the NP, the receptor density within the contact area is assumed to increase because the ligands recruit additional receptors to this area. Therefore, the optimal NP size for fast membrane wrapping is governed by the competition between the thermodynamic driving force (ligand–receptor binding) and the receptor diffusion kinetics to the internalization site was determined as 54–60 nm [46]. Thus, our 100 nm PS NPs are presumably capable of providing enough ligand–receptor binding strength to initiate internalization. As observed with our experiments (Figure 4), accumulation on the membrane is not required.

## Conclusion

By using spinning disk confocal microscopy, we have systematically investigated the in situ internalization process of NPs in the size range of 3.3–100 nm by live cells. Small DPA-QDs and AuNCs smaller than 10 nm (diameter) accumulate on the plasma membrane prior to their internalization by the cell, whereas polystyrene NPs with diameters of ≈100 nm were observed to directly internalize without prior accumulation, irrespective of their surface charges. Moreover, markedly different mechanisms were shown to be involved in the endocytosis of NPs with identical size and surface charge, but different surface functionalities.

In summary, these studies demonstrate the enormous complexity of the interaction between NPs and biological matter. For meaningful investigations, it is of utmost importance to explore the dependence on the many parameters in a systematic way. For an experiment to be meaningful, one has to keep all but one parameter fixed.

## Experimental

### NP synthesis

DHLA-AuNCs were prepared according to a microwave-assisted protocol as previously reported [29]. The as-obtained AuNCs were purified by centrifugation filtration using Nanosep filters (Pall Nanosep, Ann Arbor, MI), and re-suspended in phosphate-buffered saline (PBS, containing monobasic potassium phosphate, sodium chloride and dibasic sodium phosphate, Invitrogen, Carlsbad, CA) for later use.

DPA-QDs were prepared as previously described [28]. Briefly, CdSe/ZnS core/shell QDs were synthesized in organic solvent prior to ligand exchange with DPA, yielding water-soluble zwitterionic QDs.

All PS NPs, labeled with the fluorescent dye *N*-(2,6-diisopropylphenyl)-perylene-3,4-dicarbonacidimide (PMI, BASF, Ludwigshafen, Germany), were synthesized by a miniemulsion polymerization approach [30].

### Cell culture

HeLa cells were cultured in Dulbecco’s modified eagle medium (DMEM), supplemented with 10% fetal bovine serum (FBS), 60 µg/mL penicillin, and 100 µg/mL streptomycin in a humidified incubator (Heraeus, Thermo Fisher Scientific Inc., Germany) at 37 °C and 5% CO_2_. Cells were seeded in eight-well LabTek^TM^ chambers (Nunc, Langenselbold, Germany) and allowed to adhere overnight in a humidified incubator at 37 °C and 5% CO_2_ before they were washed twice with PBS.

Human MSCs were obtained from bone marrow aspirates or explanted hip bones [50] and cultured in alpha minimal essential medium (R-MEM, Cambrex, East Rutherford, NJ) supplemented with 20% fetal calf serum (FCS), 100 U penicillin, 100 mg/mL streptomycin, and 1 mM pyruvate (Sigma-Aldrich) in a humidified incubator (Heraeus, Hanau, Germany) at 37 °C. For confocal imaging, cells were seeded at a density of ≈10,000 cells/cm^2^.

### Confocal imaging of NP uptake

Cells were grown on eight-well chambered coverglasses (Nunc, Langenselbold, Germany) overnight (37 °C, 96% humidity and 5% CO_2_). After washing with PBS, cell membranes were stained with 0.25 µg/mL CellMask^TM^ DeepRed (Invitrogen) in PBS for 5 min and washed twice with PBS. For observing NP uptake kinetics, cells were incubated with PBS containing NPs at specified concentrations (QDs, 10 nM; anionic and CPS, 75 µg/mL; cationic and NPS, 7.5 µg/mL). AuNCs were suspended in serum-free DMEM at 20 µg/mL. Live cell imaging was performed for up to 2 h with our spinning disk laser scanning confocal microscopy systems [29,31]. Images were acquired in two separate color channels. NP emission was collected through a bandpass filter (AHF, Tübingen, Germany). Detailed information on the experimental conditions is given in Table 3. Suitable control experiments were performed to ensure negligible crosstalk between the two channels. Images were quantitatively analyzed using the software ImageJ [51]. The fluorescence intensity of NPs in each cell was obtained by normalizing the integrated intensity by the cell area. The cell membrane and the intracellular region were identified based on the membrane staining.

**Table 3:** Excitation and emission wavelengths used in the imaging studies.

NP	excitation NP (nm)	emission filter NP (nm)	excitation membrane (nm)	emission filter membrane (nm)

DHLA-AuNCs	405	685/40^a^	640	685/40^a^
DPA-QDs	532	585/80^a^	637	635 LP^b^
CPS NPs	473	585/50^a^	637	635 LP^b^
NPS NPs	532	585/50^a^	637	635 LP^b^

^a^Center wavelength/width. ^b^Long-pass filter.
